# Mortality within 30 days of chemotherapy: a clinical governance benchmarking issue for oncology patients

**DOI:** 10.1038/sj.bjc.6603498

**Published:** 2006-12-12

**Authors:** M E R O'Brien, A Borthwick, A Rigg, A Leary, L Assersohn, K Last, S Tan, S Milan, D Tait, I E Smith

**Affiliations:** 1 Department of Medicine, Royal Marsden Hospital NHS Trust, Downs Road, Sutton, Surrey SM2 5PT, UK

**Keywords:** mortality, chemotherapy, benchmark

## Abstract

No national benchmark figures exist for early mortality due to chemotherapy unlike for surgical interventions. Deaths within 30 days of chemotherapy during a 6-month period were identified from the Royal Marsden Hospital electronic patient records. Treatment intention – curative or palliative, cause of death and number of previous treatments – were documented. Between April 2005 and September 2005, 1976 patients received chemotherapy with 161 deaths within 30 days of chemotherapy (8.1%). Of these, 124 deaths (77.0%) were due to disease progression. Of the other 37 deaths, 12 (7.5%) were related to chemotherapy, six each for solid tumours and haematological malignancies, of which seven (4.3%) were due to neutropenic sepsis. For the remaining 25 deaths (15.5%) there was insufficient information. There were more deaths after third and subsequent lines of therapy than with first and secondlines of therapy. Only 12 of the 161 deaths occurred in patients who were receiving potentially curative chemotherapy to give a mortality rate in breast and gastrointestinal malignancy of 0.5 and 1.5%, respectively. It is possible to audit mortality within 30 days of chemotherapy and this should become a benchmark for standard practice nationally. Most deaths were due to disease progression in the palliative setting. We practice this form of audit each quarter and feed back to the treating teams so that deaths are discussed and practice monitored.

Over the last three decades, there has been a large increase in both the number of chemotherapy treatments available to cancer patients and in their usage. There is clear evidence of the beneficial effects of these treatments in terms of improving both survival and cancer-related symptoms. There is also considerable experience in the identification, grading and management of toxicities associated with these treatments. Despite this, chemotherapy-related deaths still occur and at the current time there is no formal monitoring of the frequency of occurrence in the UK.

Clinical trials, in general, publish death rates related to chemotherapy. However data outside the context of clinical trials are more limited. Ohe *et al* (2001) provide one of the few publications to deal specifically with treatment-related deaths following chemotherapy in routine clinical practice. The outcome of 926 patients diagnosed with lung cancer requiring chemotherapy with or without thoracic radiotherapy, at the National Cancer Centre Hospital, East Japan, was audited retrospectively. Fifty-two per cent of the patients had stage I–III lung cancer, whereas the remaining 48% had stage IV disease. The authors defined treatment-related deaths as all deaths within 4 weeks of completing treatment without clear evidence of other causes. In this series, 2.3% of patients died from chemotherapy-related toxicity and 1.6% died from acute pneumonitis following thoracic radiotherapy. Unsurprisingly, advanced clinical stage of cancer and poor performance status correlated with an increased risk of a chemotherapy-related death. Radford *et al* (1993) report a 5% mortality rate owing to sepsis following routine chemotherapy for small–cell lung cancer. Stephens *et al* (1994) found that 10% of 2196 patients with small-cell lung cancer entered into a series of six randomised clinical trials conducted by the Medical Research Council (MRC) Lung Cancer Working Party (LCWP) died within 3 weeks of the start of chemotherapy and that half of these deaths may have been treatment-related.

A retrospective study of 267 patients, aged 60 years or over, with intermediate or high-grade Non-Hodgkin's lymphoma receiving CHOP chemotherapy was conducted. This demonstrated a chemotherapy-related death rate of 13%. Sixty-three per cent of these deaths occurred after the first cycle of CHOP. Infection accounted for 82% of these chemotherapy-related deaths. Again, performance status was shown to be an independent prognostic factor for treatment-related death (Gomez *et al*, 1998).

Auditing patient outcomes relating to a particular intervention is not a new concept. In the early 1980s a confidential, anonymous review of mortality associated with anaesthesia was set up in the UK (Lunn and Mushin (1982)). The aim was to assess perioperative information in the hope of improving the anaesthesia and hence patient outcomes. In 1982, this became a joint surgical and anaesthetic venture named the Confidential Enquiry into Perioperative Death (CEPOD). Owing to its success, by 1988 this had become the National Confidential Enquiry into Perioperative Deaths (NCEPOD) with the majority of funding provided by the UK government. The first official report was published in 1990. NCEPOD was re-assessed in 2001 by the Grimley–Evans review and it was recommended that its remit be extended to include medical as well as surgical deaths regardless of whether a procedure had been performed. NCEPOD now encompasses primary care deaths and ‘near misses’ in addition to hospital in-patient events. As a result, the name was changed to National Confidential Enquiry into Patient Outcome and Death (conveniently still abbreviated to NCEPOD). Not surprisingly, this led to the collection of a large volume of data. From 1 April 2004, routine collection of deaths is no longer required, but rather specific studies are focussed on certain procedures for example, the report investigating in-patient deaths within 30 days of a therapeutic endoscopic procedure. Since April 2005, NCEPOD now falls under the auspices of the National Patient Safety Agency.

The increasing importance of clinical governance in relation to all aspects of patient care and the expansion of the role of NCEPOD led to the inception of this study. The aim was to monitor all deaths within 30 days of chemotherapy at the Royal Marsden Hospital and to identify whether deaths were treatment-related or not. The period of 30 days conveniently fits with the most common schedule for chemotherapy – a 21 or 28 day cycle, devised with the intention that blood counts have returned to normal before further treatment.

## METHODS

The Royal Marsden Hospital is a large cancer centre in London, UK. Patient records are stored electronically on the Electronic Patient Record (EPR). In-patient deaths are automatically registered on the EPR. Patient deaths occurring outside the hospital are added to the EPR as soon as the hospital team is notified. Notification can be via the GP, family, local hospice or the cancer registry. This audit was conceived in October 2003 followed by development of the process and was approved by the Royal Marsden Hospital Audit Liasion Group. The EPR database was searched for all patients who had received one or more cycles of chemotherapy between 1 April 2005 and 30 September 2005. The EPR database was also used to identify patient case notes for all patients who had died within 30 days of receiving a chemotherapy treatment. These case notes were analysed by registrars/consultant in medical oncology to identify the cause of death. When the cause of death was not clearly apparent from the case notes, the GP or hospice was contacted. Deaths were then categorised as being treatment-related, non-treatment-related or inconclusive owing to lack of information.

For the purposes of the study, the definition of chemotherapy treatment included cytotoxic drugs and biological agents, such as interferon and monoclonal antibody therapies. For those patients identified to have died within 30 days of chemotherapy, additional information was collected: specifically the patient's age, gender, primary cancer, the hospital unit providing treatment, the choice of chemotherapy regimen, the number of lines of previous chemotherapy treatment, the number of cycles of chemotherapy of the current regimen received, median time to death following chemotherapy and the place of death. Finally, for all identified deaths it was determined whether the chemotherapy was given with curative or palliative intent.

## RESULTS

Between April 2005 and September 2005, 1976 patients received chemotherapy treatment. During this 6-month period, 161 deaths within 30 days of receiving chemotherapy treatment were identified (8.1%). Table 1 demonstrates the demographic composition of the patients who died within this period of 30 days from treatment. The median age of patients who died was 61 years (range: 6–86 years). Fifty-three per cent of patients were female; 47% were male. The median time to death following treatment was 17 days (range 1–30 days). Eighty-one patients (50.3%) died while an in-patient at the Royal Marsden, 43 patients (26.7%) died in a hospice or another hospital, 23 (14.2%) at home and in 14 (8.6%) of cases, the location of death was not recorded.

Of the 161 deaths within 30 days of receiving chemotherapy, 124 deaths (77%) were considered to be unrelated to chemotherapy with the majority of deaths owing to progression of the underlying malignancy (75.6%). Other less common causes of death were non-neutropenic sepsis (16.2%), thrombo-embolism/cardiac arrest/SVCO (8.9%) and bowel obstruction (2.4%). Twelve deaths (7.5%) were considered related to chemotherapy: seven owing to neutropenic sepsis (4.3%), four associated with multiorgan failure/sepsis and one paediatric case attributed to chemotherapy toxicity related to impaired renal function. Of the 12 deaths, six of these patients were receiving treatment for a haemato-oncological disorder (lymphoma, myeloma and leukaemia). On excluding these haemato-oncology patients from the cohort, the total number of chemotherapy-related deaths was six, therefore the rate of deaths considered related to chemotherapy was 3.75% each for solid tumours and haematological malignancies (Table 2). Analysing each of the 12 chemotherapy-related deaths individually, seven of these deaths (58.3%) occurred in patients receiving potentially curative treatment, of which four patients were being treated for haemato-oncological disease. For 25 deaths (15.5%) occurring within 30 days of treatment, there was insufficient information to establish whether death was treatment-related. In some of these cases, treatment-related death cannot be ruled out.

Of the total 161 patient deaths within 30 days of receipt of chemotherapy, 12 patients were receiving treatment with potentially curative intent (i.e., neoadjuvant/adjuvant treatment) (Table 2). Looking closer at treatment within the breast unit, a total of 356 patients were treated with chemotherapy during this 6-month period: 199 patients with neoadjuvant/adjuvant treatment with curative intent and 167 patients with palliative intent. There was one neoadjuvant treatment related event in this 6-month period in the breast unit. This translates to a 0.5% mortality rate following potentially curative breast cancer treatment. Within the gastrointestinal (GI) unit, 349 patients were treated with chemotherapy during this 6-month period – 133 patients with curative intent, 216 patients with palliative intent. Two patient deaths occurred within 30 days of treatment in the cohort of patients receiving GI neoadjuvant/adjuvant treatment – a 1.5% treatment related mortality rate (Table 3).

There were 44 patients (27%) who died while receiving their first line of chemotherapy, 39 (24%) patients died after two lines of chemotherapy treatment and 72 patients (45%) were on third line, or subsequent lines of treatment (Table 4). In six cases (4%), the line of therapy was not documented.

## DISCUSSION

This audit describes results at the Royal Marsden for mortality following chemotherapy treatment with curative and palliative treatment, outside the context of a clinical trial. Like Ohe *et al* (2001) in Japan, this audit details low rates of treatment-related death – occurring in 7.5% of patients receiving chemotherapy in this particular 6-month time period (3.75% each for solid tumours and haemato-oncological disease). For chemotherapy given with curative intent, the mortality was 0.5 and 1.5% for the two common tumour types where adjuvant chemotherapy is given (breast and GI).

Following the collection of the quarterly data, deaths within this time are reviewed on an individual basis by each treating team. For example, the deaths within 30 days of chemotherapy within the breast unit for this period were individually reviewed. Thirty-two deaths occurred, 50% of these deaths attributed to progressive disease. Both of the two treatment-related deaths were due to neutropenic sepsis, one occurring in a patient receiving neo-adjuvant treatment. Notably, of the 32 deaths, 21 patients (65.6%) were receiving at least third-line treatment.

One of the weaknesses of this audit is the fact that 25 or 15.5% of the deaths has insufficient information to ascribe them to treatment or non-treatment related deaths. None of these patients were receiving adjuvant chemotherapy or treatment in the curative setting. Given that 77% of the deaths were due to progressive disease, it is probable that around 19 of these 25 were also due to progressive disease. If we ascribe the cause of death in the remaining six patients as due to treatment, the overall death on treatment rate could be 11.2%. Future audits need to focus on this area to minimise the number of deaths from unknown causes.

Nevertheless, we feel this is an audit system that could be used for oncology centres nationally.

Over 50% of those in the overall cohort were receiving second, or later, lines of treatment. This was a population of highly treated patients, with their associated morbidity. In this circumstance, where the chances of response to multiple lines of treatment are low, these benchmarking results will facilitate a more informed discussion about the mortality rate following chemotherapy and help patients to accurately weigh up the risks of treatment. A recent audit at the Royal Marsden Hospital (RMH) by Kuciejewska *et al* (2006) looked at the response rate to chemotherapy for heavily pretreated breast cancer patients. For patients receiving third-line chemotherapy for metastatic disease, who have achieved at best stable disease in previous lines of metastatic treatment, the response rate to this line of therapy is 20%, with a time to progression of 3 months and a median survival of 6 months. The results of this audit suggest that the rate of death within 30 days of treatment in this group of third line chemotherapy treatment patients is not insignificant at 12.6%. The potential fatal complications of treatment must be balanced with the low chance of benefit from treatment and require honest discussion with the patient. Incorporating performance status to the data collection may add extra weight to the decision-making process, with the assumption that patients with a poor performance status at time of chemotherapy have a greater mortality rate.

The study highlights the importance of accurate records and the need for timely and accurate discharge summaries. The death records depend on the involvement of the cancer registry and of the family/hospice route of informing the hospital about patient death. This has proved to be a useful exercise, which is in accordance with the governance of NCEPOD. Just recently, there has been a national movement to do a similar type of study with a protocol ‘Systemic Anti-Cancer Therapies (SACT)’ going through the NCEPOD steering group. Our data can contribute to creating a benchmark upon which other yearly periods, and the data from other hospitals, can be compared.

## Figures and Tables

**Table 1:** Demographics

Gender	Male: 75 (47%)	Female 86 (53%)
Age	Median 61 years (range 6–86)	
Time to death following chemotherapy	Median 17 days (range 1–30)	
Place of death	RMH	81 (50.3%)
	Hospice/other hospital	43 (26.7%)
	Home	23 (14.2%)
	Not recorded	14 (8.6%)

RMH=Royal Marsden Hospital.

**Table 2:** Cause of death

	**Division**														
**Total number of deaths identified from HIS**	**GI tract**	**Urology**	**Lung**	**Breast**	**Gynae**	**Clinical pharmacology**	**Skin and melonoma**	**Sarcoma**	**Head and neck**	**Paediatrics**	**Lymphoma**	**Myeloma**	**Leukaemia**	**Neuro-Oncology**	**Total**
*Likely cause of death*															
Progression of disease	12	2	13	16	6	2	5	10	4	2	4	4	10	3	93 (57%)
Liver failure											1				1
Thrombo-embolism/cardiac arrest/SVCO	3		4	1	2			1							11
Bowel obstruction				1	2										3
Chemotherapy toxicity related to impaired renal function										**1**					**1**
Sepsis (non-neutropenic)/multiorgan failure/GVHD	4	1	3	3				1	2	1		1	**4**		20
Neutropaenic sepsis	**0**	**0**	**1**	**2**	**(1)**			**1**	**0**	**0**	**0**	**1**	**1**		**7 (4%)**
															
*Summary of deaths*															
Unknown (Insufficient Information)	1	2	3	9	6	0	3	1	0	0	0	0	0	0	25 (16%)
Total chemotherapy unrelated deaths	19	3	19	21	10	2	5	12	6	3	5	5	10	3	124 (77%)
** Total chemotherapy related deaths**	**0**	**0**	**2**	**2**	**(1)**	**0**	**0**	**1**	**0**	**1**	**0**	**1**	**5**	**0**	**12 (7%)**

GI=gastrointestinal; GvHD=graft-versus-host disease; SVCO=superior vena cava obstruction.

**Table 3:** Curative treatment mortality rate – breast/GI

**Treatment intent**	**Breast unit**	**GI unit**
Neoadjuvant/adjuvant	199 (54%)	133 (38.1%)
Palliative	167 (46%)	216 (61.9%)
Total	366	349
		
Neo/adjuvant deaths	1 (0.5%)	2 (1.5%)

GI=gastrointestinal.

**Table 4:** Lines of treatment

	**Division**														
**Total number of deaths identified from HIS**	**GI tract**	**Urology**	**Lung**	**Breast**	**Gynae**	**Clinical pharmacology**	**Skin and melonoma**	**Sarcoma**	**Head and neck**	**Paediatrics**	**Lymphoma**	**Myeloma**	**Leukaemia**	**Neuro-Oncology**	**Total**
Total no. of deaths	20	5	24	32	17	2	8	14	6	4	5	6	15	3	161
Patients on 1st line therapy	11	2	11	3	3	0	4	4	0	2	0	1	2	1	44 (27%)
Patient on 2nd line therapy	1	0	6	7	3	0	3	8	6	1	1	0	2	1	39 (24%)
Patients on 3rd line or subsequent therapy	7	3	6	21	10	2	0	2	0	1	4	5	10	1	72 (45%)
Line of therapy not known/not applicable	1	0	1	1	1	0	1	0	0	0	0	0	1	0	6 (4%)
Total no. of deaths related to neo-adjuvant/adjuvant treatments	2	0	0	1	0	0	0	0	1	1 (1)	0	2	4	0	12
